# Acute Care Research Requires an Adapted Consent Procedure to Safeguard Participants' Autonomy and Rights While Limiting the Risk of Consent‐Bias

**DOI:** 10.1111/acem.70233

**Published:** 2026-01-23

**Authors:** Sywert O. Westerhof, Carolina Hincapié‐Osorno, Raymond J. van Wijk, Ewoud ter Avest, Barbara C. van Munster, Jan C. ter Maaten, Hjalmar R. Bouma

**Affiliations:** ^1^ Department of Acute Care, University of Groningen University Medical Centre Groningen Groningen the Netherlands; ^2^ Department of Internal Medicine, University of Groningen University Medical Centre Groningen Groningen the Netherlands; ^3^ Grupo de Investigación en Urgencias y Emergencias (GIURE) Universidad de Antioquia Medellín Antioquia Colombia; ^4^ London's Air Ambulance Charity London UK; ^5^ Department of Clinical Pharmacy and Pharmacology University of Groningen, University Medical Centre Groningen Groningen the Netherlands

**Keywords:** acute care, Acutelines, consent bias, informed consent, participation bias

Acute medicine is characterized by its unpredictable nature. In contrast to other clinical research settings, it is usually not possible to identify candidates for research prior to the event that triggers the inclusion of the participant in a clinical study. Patients may be severely ill, have an impaired level of consciousness or delirium, which can affect their ability to provide informed consent. Strictly relying on informed consent by the patient may result in participation bias [1, 2, 3, 4]. On the other hand, research data collection requires a legal ground, which is usually consent or (by exemption) public interest [5, 6, 7].

To study the correlation between severity of illness and the ability to provide consent, with relevance to participation bias in acute research, we performed a post hoc analysis of patients prospectively included in the Acutelines data‐biobank [6] at the University Medical Center Groningen where we introduced an adapted, stepped consent procedure that is GDPR (General Data Protection Regulation) compliant and approved by the institutional research review board. If possible, consent for inclusion of data and biomaterials in the data‐biobank was obtained directly from the participant within 24 h (direct consent). If this was not feasible, deferred consent was sought from the participant within 30 days. When the participant remained unable to provide consent, consent by proxy was obtained within the same timeframe. If all options were exhausted, data and biomaterials could be included under an opt‐out procedure after confirming in the UMCG objection registry that the participant had not objected to research use of their data or samples. In all cases, patients or relatives were informed orally by a nurse or researcher and received a patient information leaflet during hospitalization; participation was recorded and visible to the participant via the MyUMCG health record app. Participants retained the right to withdraw at any time without providing a reason, and could request destruction of their data and materials [6, 7].

To assess participation bias by type of informed consent, patient characteristics were stratified by consent type: direct, deferred, by proxy, and opt‐out. Categorical variables were reported as counts and percentages, and continuous variables as means with standard deviations or medians with interquartile ranges, depending on distribution. Normality was assessed using *Q–Q* plots and the Shapiro–Wilk test. Between‐group differences were analyzed using the Kruskal–Wallis test for continuous variables, followed by Mann–Whitney *U* tests for pairwise comparisons with Benjamini–Hochberg correction for multiple testing. For categorical variables, chi‐squared tests were applied. Differences in mortality were visualized using Kaplan–Meier curves and analyzed with the Cox proportional hazards model. A *p*‐value < 0.05 was considered statistically significant. Data processing and analysis were performed using Stata 18 and R version 4.2.2.

For this post hoc analysis, we included 1860 adult patients (≥ 18 years) who were admitted to the emergency department (ED) for gastroenterology, internal medicine (including geriatrics, nephrology, oncology, hematology), pulmonology, rheumatology, emergency medicine (nontrauma), or urology between May 5, 2022, and September 1, 2023. Repeated ED visits were excluded. Of these, 1043 patients (56%) provided direct consent within 24 h, 185 (10%) deferred consent within 30 days, 167 (9%) consented by proxy, and 465 (25%) participated via opt‐out. Patients with consent by proxy (median age 73, IQR 62–82) were on average older than those who provided direct consent (68, IQR 57–76), deferred consent (68, IQR 57–75), or participated by opt‐out (61, IQR 52–70).

To assess whether disease severity is associated with the type of consent, patients were stratified by the modified early warning score (MEWS) measured upon arrival to the ED: MEWS 0–1 (*n* = 1091, 59%), MEWS 2–4 (*n* = 603, 32%), and MEWS ≥ 5 (*n* = 118, 6%). Among the least severely ill patients, who had a MEWS of 0 or 1, most patients provided direct consent, followed by opt‐out, deferred consent, and consent by proxy. The same pattern was seen for patients with MEWS between 2 and 4. Among the most severely ill patients with MEWS ≥ 5, however, participation by opt‐out was most frequent, followed by direct, by proxy, and deferred consent approaches (Figure 1a).

**FIGURE 1:**
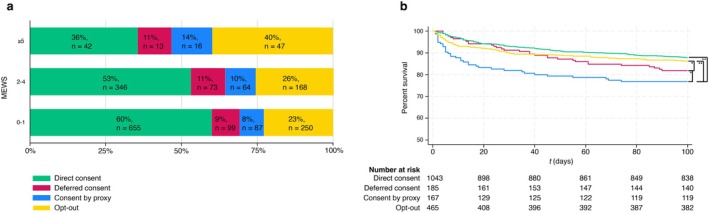
Overview of consent type by disease severity and survival. Kaplan–Meier survival by type of consent. (a) Distribution of consent types across MEWS strata. (b) Kaplan–Meier curves showing 90‐day survival and number at risk per consent type. Significant differences based on Cox proportional hazards analysis are indicated (*p < 0.05; **p < 0.005).

During hospitalization, 140 patients (8%) were admitted to the Intensive care unit (ICU). As compared to patients who provided direct consent, where ICU admission occurred in 42 patients (4%), ICU admission was more common among patients who provided deferred consent (*n* = 25, 14%, HR 2.02, 2.11–4.72, *p* < 0.005). ICU admission was not different between patients who provided (95% CI 1.20–3.39, *p* < 0.005) and those who participated by opt‐out (*n* = 16, 10%, HR 3.16, 95% CI) direct consent and those who provided consent by proxy (*n* = 57, 12%, HR 1.63, 95% CI 0.90–2.95).

The average 90‐day survival rate was 93%: a total of 128 patients died. Patients who provided direct consent, had a 90‐day survival rate of 94%, whereas this was 91% for patients in whom deferred consent was obtained, 86% for patients where consent was provided by proxy, and 94% for patients who participate by opt out (*p* < 0.005). The 90‐day survival rate was not different between patients who participated by opt‐out (94%) as compared to those who provided consent themselves (Figure 1b). Compared with direct consent, both deferred consent and opt‐out were associated with higher rates of ICU admission.

Our findings demonstrate that disease severity, and outcomes are related to the type of informed consent provided. Patients are less likely able to provide consent themselves, either direct (at the ED) or deferred within the 30 days following ED admission. A study‐recruitment strategy using only direct or deferred consent will therefore introduce participation bias resulting in underestimation of ICU admission rates and 90‐day survival. The stepped consent procedure that we have introduced in the Acutelines data‐biobank may prevent this, as it aims to safeguard the autonomy and rights of the participants, while limiting participation bias. We consider this stepped consent procedure preferable over a simple opt‐out approach, as opt‐out alone does not ensure that patients or their representatives are adequately informed about study participation and data use. In addition, an opt‐out procedure may undermine the ethical principle of informed consent, particularly in acute settings where patients are temporarily incapacitated. The stepped consent procedure balances ethical transparency and participant autonomy with the practical need to include severely ill patients, thereby reducing selection bias while maintaining compliance with ethical and legal standards.

The stepped consent procedure, which includes consent by proxy and opt‐out in exceptional cases where participants cannot provide consent themselves, aligns with European legislation [8] that allows exemptions for reasons of public interest under the GDPR and the European Health Data Space [9]. While opt‐out procedures facilitate inclusion of acutely ill patients in research, the GDPR continues to restrict the exchange of personal and pseudonymized data between institutions, as these remain subject to data protection requirements and typically require consent from the participant or their representative. True anonymization, which would remove these restrictions, is rarely achievable in biomedical research because data such as clinical variables, laboratory results, or imaging often remain indirectly identifiable. Emerging approaches such as data synthesis or federated learning may offer alternatives, allowing analyses across institutions without direct data exchange, but these methods are still developing and not yet widely accepted for regulatory or clinical use.

In conclusion, our findings show that relying solely on direct informed consent in acute care research may result in participation bias. Implementing a stepped consent procedure involving participation via consent by proxy and opt‐out may prevent this, as it allows the inclusion of a study population that more accurately reflects the real‐world emergency department population, thereby improving the validity and generalizability of research outcomes to clinical practice.

## Author Contributions

Conceptualization and design: H.R.B., S.O.W., E.A., B.C.M., J.C.M. Data acquisition and analysis: S.O.W., R.J.W., C.H.‐O. Data interpretation: S.O.W., C.H.‐O., R.J.W., H.R.B., J.C.M. Writing: S.O.W., C.H.‐O. Review: R.J.W., H.R.B., E.A., B.C.M. Supervision: H.R.B., J.C.M.

## Funding

This research has been funded by the University Medical Center Groningen. The establishment of Acutelines has been made possible by funds from the University Medical Center Groningen.

## Ethics Statement

This study was approved by the Central ethics Review Board non‐WMO studies of the University Medical Center Groningen (registration number: 18425).

## Conflicts of Interest

The authors declare no conflicts of interest.

## Data Availability

Data used in this study can be obtained upon reasonable request via the Acutelines data and biobank. https://doi.org/10.34760/5f5b7d8a064c8.
